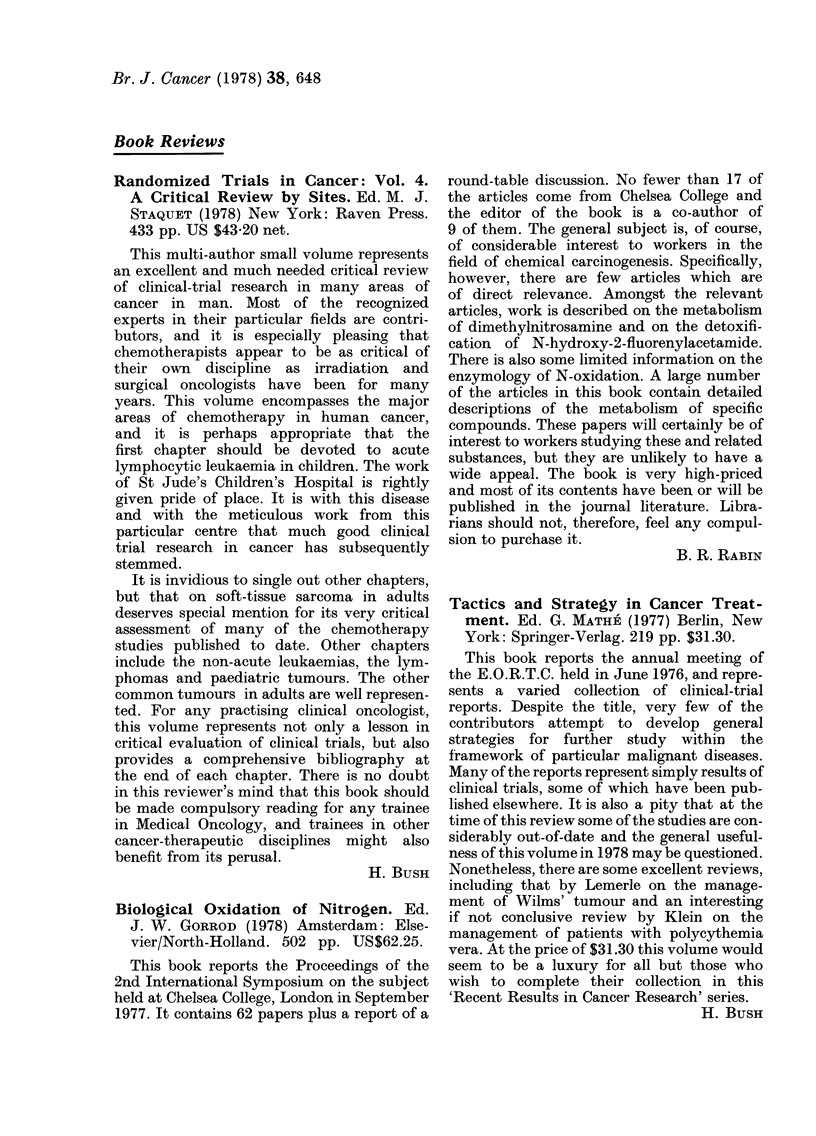# Biological Oxidation of Nitrogen

**Published:** 1978-11

**Authors:** B. R. Rabin


**Biological Oxidation of Nitrogen.** Ed. J. W. GORROD (1978) Amsterdam: Elsevier/North-Holland. 502 pp. US$62.25.

This book reports the Proceedings of the 2nd International Symposium on the subject held at Chelsea College, London in September 1977. It contains 62 papers plus a report of a round-table discussion. No fewer than 17 of the articles come from Chelsea College and the editor of the book is a co-author of 9 of them. The general subject is, of course, of considerable interest to workers in the field of chemical carcinogenesis. Specifically, however, there are few articles which are of direct relevance. Amongst the relevant articles, work is described on the metabolism of dimethylnitrosamine and on the detoxification of N-hydroxy-2-fluorenylacetamide. There is also some limited information on the enzymology of N-oxidation. A large number of the articles in this book contain detailed descriptions of the metabolism of specific compounds. These papers will certainly be of interest to workers studying these and related substances, but they are unlikely to have a wide appeal. The book is very high-priced and most of its contents have been or will be published in the journal literature. Librarians should not, therefore, feel any compulsion to purchase it.

B. R. RABIN